# Visualizing the Landscape of Home IoT Research: A Bibliometric Analysis Using VOSviewer

**DOI:** 10.3390/s23063086

**Published:** 2023-03-13

**Authors:** Jue Wang, Hak-Seon Kim

**Affiliations:** 1 School of Global Studies, Kyungsung University, 309 Suyoungro, Nam-gu, Busan 48434, Republic of Korea; 2 School of Hospitality & Tourism Management, Kyungsung University, 309 Suyoungro, Nam-gu, Busan 48434, Republic of Korea; 3 Wellness & Tourism Big Data Research Institute, Kyungsung University, 309 Suyoungro, Nam-gu, Busan 48434, Republic of Korea

**Keywords:** home IoT, bibliometric analysis, trends, VOSviewer

## Abstract

Currently, the internet of things (IoT) is being widely deployed in home automation systems. An analysis of bibliometrics is presented in this work that covers articles that were obtained from the Web of Science (WoS) databases and published between 1 January 2018, and 31 December 2022. With VOSviewer software, 3880 relevant research papers were analyzed for the study. Through VOSviewer, we analyzed how many articles were published about the home IoT in several databases and their relation to the topic area. In particular, it was pointed out that the chronological order of the research topics changed, and COVID-19 also attracted the attention of scholars in the IoT field, and it was emphasized in this topic that the impact of the epidemic was described. As a result of the clustering, this study was able to conclude the research statuses. In addition, this study examined and compared maps of yearly themes over 5 years. Taking into account the bibliometric nature of this review, the findings are valuable in terms of mapping processes and providing a reference point.

## 1. Introduction

As a term used to refer to a network of connected devices and systems, the concept of the internet of things (IoT) encompasses a vast range of technologies, devices, and services that can be accessed anywhere on the planet, anytime, and was associated with many different fields [1]. The internet of things and smart homes gave rise to a new domain, namely, smart home IoT [2]. As discussed by Ejaz et al. (2007), the IoT is a rapidly expanding application area that can reduce the latency in communication in the home and provide residents with a better quality of life [3]. A smart building can reduce the impact of intrusion, enable proactive and intelligent actions, and enhance the quality of life by managing data in a proactive and intelligent manner, which will change all domains by providing real-time information from interconnected devices at any moment, anywhere, anytime, on any device. There is a growing interest in home IoT in both the academic and technical communities, as well as how to address, adapt, and improve the service. Furthermore, due to the COVID-19 pandemic, most tech companies forced their employees to work from home. It was thus found that over the past couple of years, several descriptive research papers were published by scholars about the application of IoT in homes.

It is worth noting that there are some papers that present the architectures of IoT of homes [1], as well as some scholars who contributed to the development from a theoretical perspective. As one of the most important tools used to assess and analyze academic research output, bibliometrics can contribute to a better understanding of the research output by allowing a reviewer to evaluate the progress made, identify the most reliable sources for scientific publications, lay the academic foundation for evaluating new developments, identify the major actors in science, etc. [4]. A significant amount of concern was aroused by the bibliometric methodology in research publications in the area of IoT. Ohlan and Ohlan (2022) examined several academic trends in the field of smart homes, as well as the intellectual structures of smart homes research from multiple perspectives in order to develop a holistic framework for the field [5]. Their results show that smart home studies grew tremendously in both volume and impact. A bibliometric analysis of smart city publications was conducted by Szum (2021) [6]. Five main areas of research were identified by the author: smart city applications, smart city architecture, energy, privacy, and security. Choi et al. (2021) utilized a bibliometric approach and analyzed research articles by obtaining papers from recognized international conferences and respected journals about smart homes and IoT [2]. Using this approach, there is the advantage of being able to summarize existing evidence relevant to the technology in question, and it enables gaps in the existing research to be identified in order to further advance the field in the future [7].

Despite this, the fact remains that it is not always clear to all of the people out there what technologies are commonly used for IoT applications in the home in the present day due to the rapid growth of new technologies and research that poses a lot of problems in managing, controlling, and enhancing IoT in the home. Thus, it is relevant to note that we have not yet been able to evaluate the available features of home IoT applications or the gaps that exist in these features. By scrutinizing the relevant home IoT literature, it will be possible to shed light on the current theoretical and practical features of the IoT in homes. In order to provide insight into this research area, studying the knowledge structure and citation landscape related to home IoT was the objective of this study. A global perspective was taken on the growth trends, the fundamental scientific indicators, the publication features, and the core journals in this field from a scientific standpoint. In addition, it explored the inner structure of home IoT research publications using bibliographic data taken from the Web of Science (WoS) database to gain a better understanding of the research process. It was intended that this study would be able to provide comprehensive results based on the use of data that were searched for across all subscribed resources and databases on the WoS.

The rest of the study was framed by the following research objectives:

(1) Providing an overview of the scientific literature concerning the home IoT sector.
(2) Considering the authorship, citations, and geography when analyzing contributions.
(3) Analyzing collaborations to identify influential organizations and authors.
(4) Identifying yearly trends in the home IoT field and evaluating their development.

**Figure 1:**
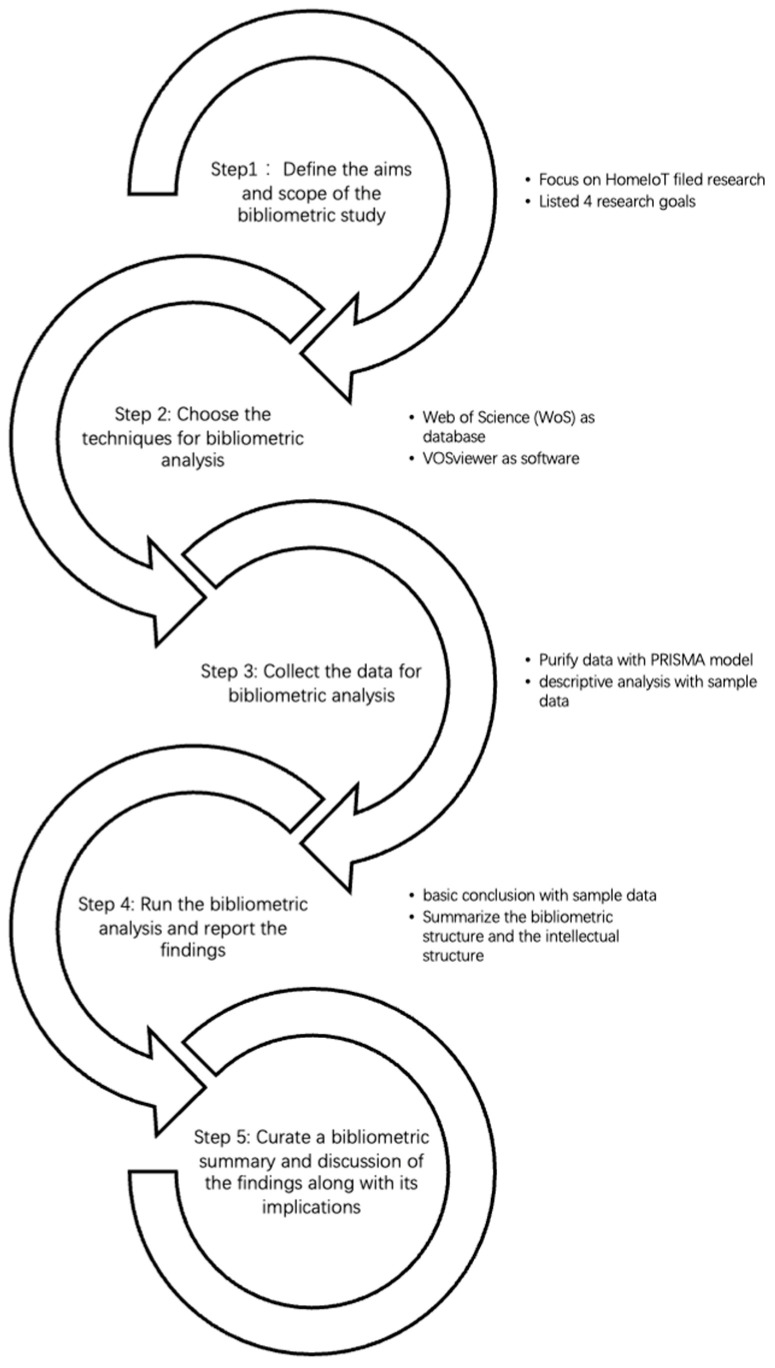
The bibliometric analysis procedure.

The flow of the study was organized as shown in Figure 1. According to Donthu (2021), there are five steps for conducting bibliometric analysis [8]. With guidelines, these are the specific sections of the study that were examined. In step 1, it was important to identify the objectives and scope of the study. As mentioned previously, this study focused on the field of home IoT, and four goals were outlined. The following literature review provides additional research background for the aims and scope of the study. A description of steps 2 and 3 can be found in the Materials and Methods section. The findings of step 4 are discussed in the section entitled Results. This work was intended to provide researchers and developers interested in home IoT with a solid foundation for further research and practical guidance going forward. Thus, as part of step five, a discussion and conclusion is presented.

## 2. Literature Review

### 2.1. Home IoT

Home automation and remote control are made possible by smart information technology installed in smart homes. Home IoT is primarily concerned with improving occupant well-being [9]. While high-speed Internet spread widely in the late 1990s, home IoT did not start being installed until the late 2000s, around the time smartphones became popular. The IoT for homes has been defined under multiple names, including home networks, digital homes, automated homes, intelligent homes, and smart homes. Home IoT that is situation-aware has been on the rise since the mid-2010s [10]. Scholars have summarized the phases of home automation and home networks from the 1990s to the 2000s to the 2010s, stating that the phase shifted from “home automation” to “smart home”. The technical background was developed that covered broadband internet, smartphones, apps, the IoT, and information technology. The background in technology ranges from broadband Internet and smartphone/apps to the IoT and information technology. In conjunction with this, the main function of a home automation system has evolved from household automation to “remote monitoring” and, in today’s world, has shifted to “context awareness” [10].

From the perspective of existing academic research, the definition of the existing home IoT is mainly from the technical and services aspects [7]. From a technical standpoint, experts believe that if a network of sensors and infrastructure are controlled by a network of sensors, homes will be able to predict and effectively respond to residents’ needs. In addition to this, the definition of smart homes also takes into account the different service objectives of these homes [7]. While defining and scoping the internet of things in homes, social aspects are also taken into account since technological advancements always affect the social acceptance of new norms [11].

It is widely accepted in the 21st century that a home internet of things device is capable of automatically monitoring and controlling appliances and devices from the outside. A typical IoT service in a home consists of lighting, power control, access, air conditioning, air quality monitoring, appliances, smart kitchen, timing, and healthcare, all of which can be extended in a way to include elder care, quality of life, comfort, home appliances, home robotics, control, home protection, security, ecology, energy conservation [11]. 

There were previous studies on home IoT that have taken a technical approach or a partial approach. For instance, in an empirical approach, there exists a relationship between the critical factors influencing the adoption behavior and the critical features that current users require. Yang et al. (2018) examined the critical factors that influence adoption behavior with 216 samples of Korean homeowners [12]. It was also tested whether personal characteristics have a moderating effect on behavior. Specifically, the study identified the specific factors associated with the adoption of smart home services, as well as analyzed the empirical relationship between acceptance behavior and the specific factors. Recently, a study by Zielonka et al. (2022) provided a snapshot of prevailing trends in homes that use IoT. The prevailing trends were healthcare and quality of life, security of data management, optimal energy management, and sustainability [11]. In a technical manner, as reviewed [13], IoT devices, such as Google Home, Amazon Echo, Amazon Firestick, and Alexa, can be used for entertainment, in addition to providing features like smart home facilities, such as Alexa and Google Assistant, through the creation of smart home devices [11,14]. Moreover, with the COVID-19 background in place, it is evident that the well-proven IoT strategy is now becoming more explored regarding providing healthcare and medical services. It is mainly referred to as the internet of medical things and is attracting the attention of many stakeholders [15]. It is believed that this technology could be a game changer in the field of orthopedics worldwide, leading to people practicing the field in a more efficient manner. The concept of the IoT in homes has become a hot topic of research due to its quality services, even in these tough economic times.

### 2.2. Bibliometric Analysis Regarding IoT Applications

A bibliometric approach is presented here in order to gain a deeper understanding of the intellectual structure and dynamics of research. A bibliographical analysis is a type of quantitative analysis of a book, an article, or another type of publication [8]. As a result of bibliometric analysis, different professional fields were able to visualize the current knowledge status, attributes, development, and trends [16]. Scholars without expertise in these fields will be able to gain a full understanding of these fields by using this method [17]. In the IoT field, scholars have offered several fruitful research outcomes. Based on the data from the WoS, the following Table 1 gives a summary of bibliometric research on home IoT. There are 10 articles listed in Table 1, arranged according to a query result obtained by searching “home IoT” or “homes IoT” or “homes” and “IoT” and “bibliometric” in the WoS database, and sorted in order of high to low citations (11 publications from all databases found, 1 failed retrieval). The field was spread across research areas such as computer science, engineering, telecommunications, materials science, other science and technology topics, communication, energy fuels, environmental sciences and ecology, health care sciences services, information science and library science, instruments and instrumentation, mathematics and medical informatics. In the database of articles, four out of ten of them used Scopus, while the remaining articles used either the WoS core alone or in combination with other databases offered by the WoS website. In terms of software, there were five articles that were not specified. Apart from this, one of the most commonly used software is VOSviewer and CiteSpace. 

There is one point to emphasize, and that is from the results in Table 1, it is clear that bibliometrics is not mature in the field of home IoT since the topic does not fully fit the scope of home IoT research. For instance, Guo et al. (2021) conducted bibliometric analysis focusing on blockchain and they mentioned smart homes as one cluster that focused on this technology [17]. Sadoughi et al. (2020) concluded that the majority of medical IoT studies and prototypes were experimental and prototypical in nature, and they generally reported that the home is the most popular place to use medical IoT applications for medical purposes [15]. An analysis of the bibliometric data of articles relating to Blockchain is presented by Karmen, and he concludes that a set of four areas should be taken into consideration as future directions: security, computing, application, and cryptocurrency [18]. A study by Rani et al. (2021) focused on security and privacy issues at different levels of smart city architecture. Home IoT is one of the domains that were discussed during this study [19]. Based on the findings of the previous study, as well as the perspective of the researchers, the IoT is widely used in a variety of applications [20]. There has been an increasing tendency among scholars to integrate IoT utilizations systematically based on their nature.

## 3. Materials and Methods

### 3.1. Materials

The important stage of a bibliometric analysis is to choose the most appropriate data source that is compatible with the scientific consensus in our research area. This will ensure that the bibliometric analysis can be done efficiently. As a matter of fact, it is imperative to emphasize the fact that there are many bibliographic databases available. Among the most popular ones are WoS, Google Scholar, SpringerLink, Embase, and many more.

**Figure 2:**
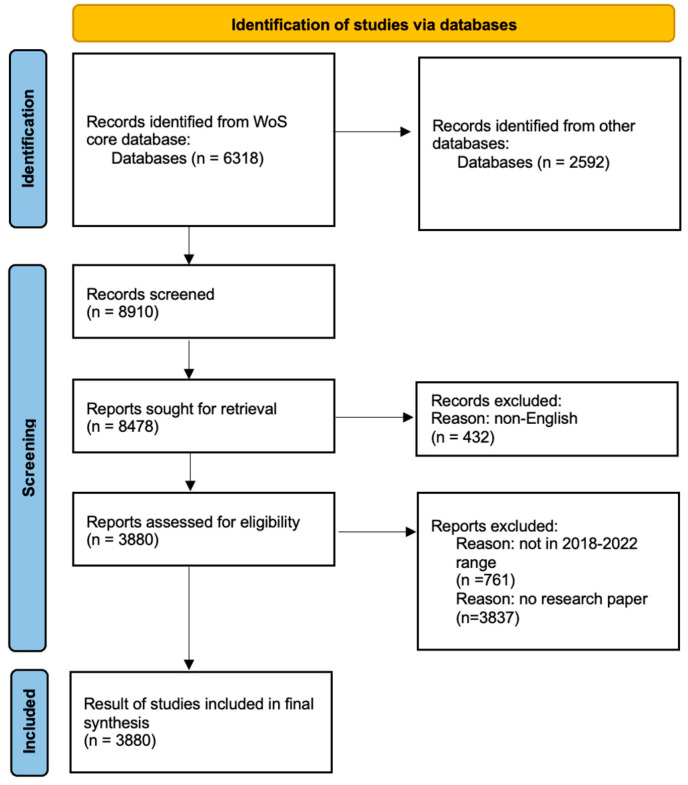
PRISMA flowchart.

Besides the core collection database of WoS, this study included all databases in the WoS website (extracted on 9 January 2023), as well as Derwent innovation indexes, MEDLINE, the KCI-Korean Journal Database, the Chinese Science Citation Database, the BIOSIS Citation Index, the SciELO Citation Index, and the Food Science Resource (FSTA). The studies were selected using the Preferred Reporting Items for Systematic Reviews and Meta-Analysis (PRISMA) flowchart and guideline [23] as a filtering tool to select the papers from the WoS database. As shown in Figure 2, this bibliometric review progressed through the identification, screening, and inclusion of papers.

We used the terms “home IoT” or “homes IoT” or “homes” and “IoT” in order to identify the closest matching publication. In total, 6318 publications were identified regarding home IoT in the WoS core database and 2592 publications were identified in the other databases without any time limitations.

Using the latest 5 years as the period in question, the global literature about home IoT that was published between 2018 and 2022 was compiled for retrieval. As a starting point, a previous study claimed that 2008–2014 was the embryonic stage of home IoT, while 2014–2017 was the fusion stage. There has been an enormous increase in the amount of research being conducted in this field since 2018 [7]. Furthermore, with the intention to figure out whether the COVID-19 pandemic has had an impact on the research progress in the field of home IoT, this study examined a period spanning two years before (2018–2020) and after the pandemic (2020–2022), as well as conducting annual time series observations.

There were several factors to be considered when processing the information for the documents that met the requirements, including the year of publication, the language, the journal, the title, the author, the affiliation, the keywords, the type of document, the abstract, and the number of citations, which could be exported into a CSV file. In consideration of these issues, this study excluded 432 publications that were not written in English in the screening process. Additionally, there were various types of documents related to a meeting or article selected for extraction. Patents, review articles, books, editorial material, unspecified, abstracts, and other unsuitable document types are eliminated (3837 records excluded).

Upon filtering, we were able to find 3880 papers that were used to conduct the analysis of this study. According to the data, the total amount of time cited was 24,166; without self-citations, this value was 21,985. Furthermore, the average number of citations per item was 6.43, while the h-index of the collection was 57.

### 3.2. Method and Software

There has been an explosion of interest in bibliometric analysis in the field of business research in recent years, largely due to advances in bibliometric software and scientific databases, and also due to the cross-disciplinary nature of the relationships between information science and business [8]. A bibliometric study provides an explanation of the intellectual structure and conceptual framework of a particular field [24]. It was suggested by Mukherjee that bibliometric research can be conducted in several different domains, for example, for a construct, a discipline, a method, or a theory [25].

It is commonly known that performance analysis and science mapping are two mainstream methods of bibliometric research. It is possible to identify social dominance or hidden bias in a field based on publication metrics by measuring the productivity of its constituents. A scientific map can be used to discover relationships between authors, institutions, or countries [8,26].

A series of bibliometric tools and techniques were used to analyze the data in this study in accordance with previous studies. After the performance analysis and the science map were completed, this study added the network analysis to the process as a step toward completion.

By using scientific maps to represent the relationship between the different actors, bibliometrics analysis can be enhanced. It shows the physical proximity and relative locations of disciplines, fields, specialties, papers, and authors in relation to one another in accordance with a map of science. The most relevant software was analyzed several times in the last few years. Researchers conducted a review that aimed to provide a comprehensive review of the various tools available for conducting bibliometric and scientometric analyses [16]. For instance, some software tools for conducting science mapping include Biblioshiny, BiblioMaps, and CiteSpace. However, a wide range of thematic, cartographic, and cluster analyses can be performed using VOSviewer, especially regarding bibliometric analysis [16]. As mentioned by researchers, VOSviewer is a tool for building and visualizing bibliometric networks based on co-citations, bibliographic couplings, and co-authorship relationships between journals, researchers, and individual publications [8,16,17].

## 4. Results

### 4.1. Publication Performance

**Figure 3:**
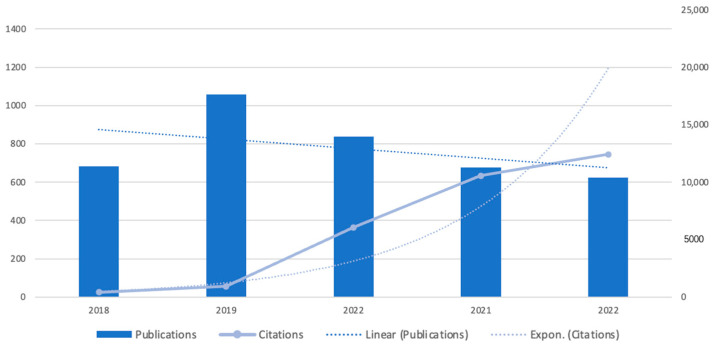
Publications and citations.

The studies were selected using the PRISMA flowchart as a filtering tool to select the papers from the WoS database. Figure 3 shows the distribution of the sample on an annual basis. On the whole, international literature grew at a steady rate in recent years on a global scale. In 2019, there were 1059 publications and 6064 citations in the field, making it the year with the most publications ever.

It is interesting to note that Choi et al. [2] pointed out that while the number of articles relating to smart homes remained constant between 2015 and 2019, the number of articles covering publications in the field of smart homes increased by 4.5 times between 2015 and 2019. However, according to Figure 3, there were several reasons why this could be the case, such as the COVID-19 pandemic or the widespread use of the IoT in other fields of business.

The top 10 most active regions, research areas, and organizations of home IoT publications are listed in Table 2. Furthermore, the WoS core collection provides MeSHs, which refers to medical subject headings from MEDLINE/PubMed. Most frequently, the MeSHs were humans (96 counts), internet of things (56 counts), aged (27 counts), monitoring physiologic (27 counts), and delivery of health care (22 counts). Clearly, the application of the IoT is geared toward the needs of the people, as can be seen from the MeSH headings provided.

### 4.2. Keywords

**Figure 4:**
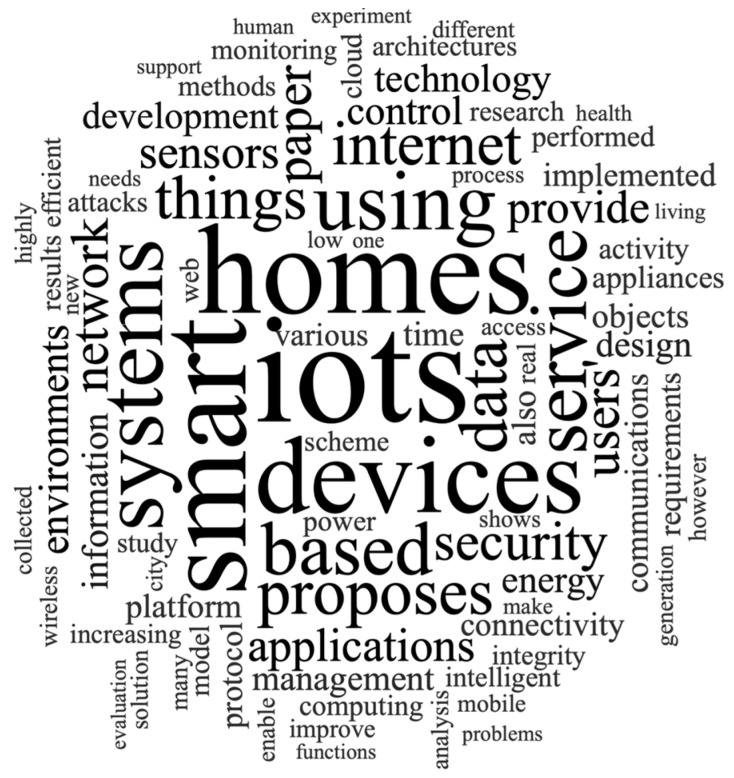
Word cloud top 100.

In the first analysis process, keywords that occurred with a high frequency in the WoS databases were provided by the authors of the paper. In terms of frequency, the most frequent keyword was “iots” with a 2.52% weighted percentage, the most similar keyword is “iot”, and the second most frequent keyword was “homes” with a 2.11% weighted percentage. The following are the next highest ranked keywords: “smart” (2.04%), “devices” (1.76%), “systems” (1.45%), “using” (1.45%), and “service” (1.21%). Figure 4 indicates the word cloud top 100.

### 4.3. Co-Authorship Analysis

Co-authorship analysis provides insight into the cooperative mechanisms between individuals and organizations that contribute to the success of science and technology partnerships [27]. Co-authorship is an official statement that more than one author or organization contributed to a technical document. Science collaboration patterns are still assessed and understood using co-authorship analysis, despite the debates about its meaning and interpretation [27,28].

**Figure 5:**
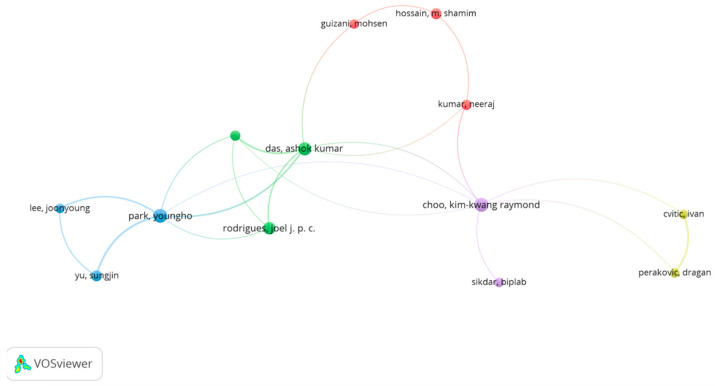
Co-authorship map of authors.

**Figure 6:**
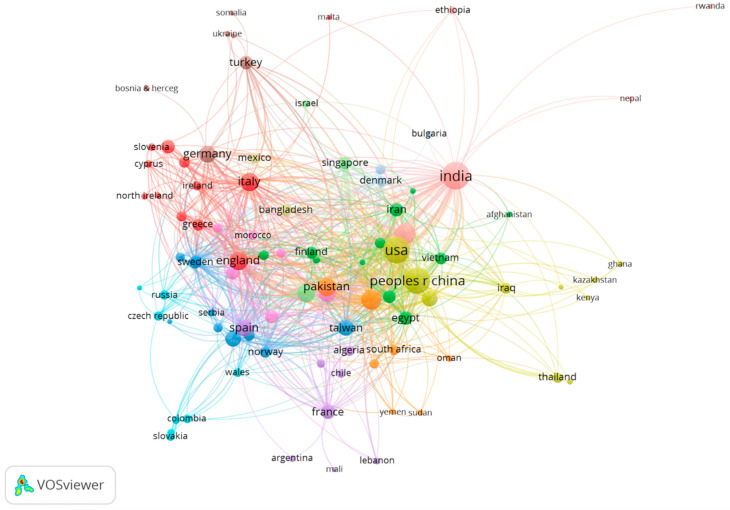
Co-authorship map of countries.

**Figure 7:**
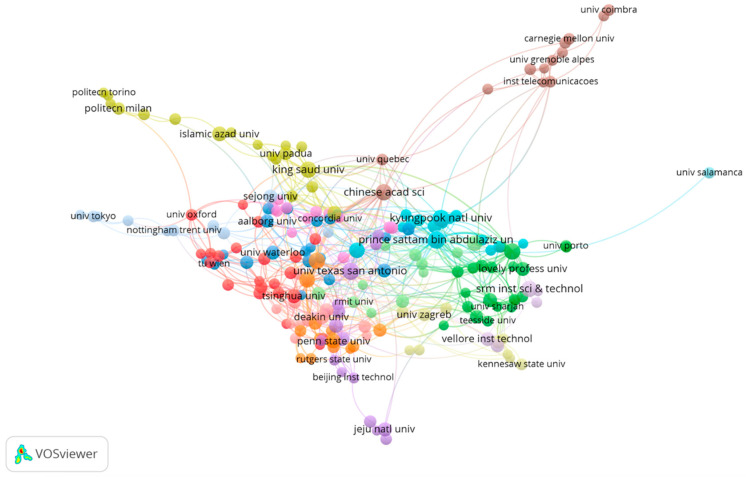
Co-authorship map of organizations.

The co-author network consisted of nodes that were assigned to them, representing authors (see Figure 5), countries (see Figure 6), or organizations (see Figure 7) that share co-authorship.

Based on the co-authorship map shown in Figure 5, there were 12,378 authors, of which 62 met the threshold of having at least 5 papers published. In terms of the total link strength, Youngho Park (15 documents, 130 citations, 24 total link strength) had the most influence, Ashok Kumar Das (12 documents, 264 citations, 20 total link strength) had the second most influence, and Kimkwang Choo (15 documents, 262 citations, 9 total link strength) had the third most influence.

In terms of the co-authorship map of countries, it is important to note that 71 of the 107 countries on the co-authorship map of countries met the minimum threshold of five documents for co-authorship. Based on Figure 6, the number of documents provided by the USA was 588, and the number of citations was 2586 based on the number of documents provided. The total link strength was 266. There were 567 documents related to the People’s Republic of China, which were cited by 3046 other documents. The total link strength was 298. A total of 465 documents and 2267 citations were cited in India as the third major country. Together, these three countries showed a strong sense of partnership.

For the co-authorship map of organizations, it is important to note that 227 of the 3085 countries in the co-authorship map of organizations met the threshold of five documents. Figure 7 shows the organizations with the greatest total link strength in the center of the map. Furthermore, the diameter of a node was directly proportional to the number of co-authorship links connecting the other modes. Thus, this approach was useful for intuitively analyzing the contribution and closeness of major research institutions.

According to the data, Prince Sattam Bin Abdulaziz University had the strongest total link strength (39), while the Chinese Academy of Sciences had the second strongest total link strength (38) among all universities. University of Texas at San Antonio was the institution with the highest number of citations (326 citations). In addition to this, it can also be observed that the most active cluster of research relationships revolved around “Beihang University”, “Cardiff University”, “Carleton University”, “Dongguk University”, “Fudan University”, “Guangzhou University”, “Hongkong Polytech University”, “Nanjing University”, “National University of Science and Technology”, “Newcastle University”, “Peng Cheng Lab”, “Shenzhen University”, “Southern University of Science and Technology”, “Stevens Institute of Technology”, “Swinburne University of Technology”, “Temple University”, “Tsinghua University”, “University North Texas”, “University Oxford”, “University South Carolina”, and “Xi’an Jiaotong University” (13 institutions in total). This led us to conclude that highly educated researchers are working on the development of the IoT in homes worldwide.

### 4.4. Co-Occurrence Analysis

As well as guiding science policies in the field of information management, word co-occurrence maps can also be useful tools for understanding the state of existing knowledge, as well as assisting in the development of specific recommendations or policies [28]. The analysis of literature keywords provides a means to determine the knowledge structure in a particular research field, as well as a means of exploring development trends within the field as a whole [29].

**Figure 8:**
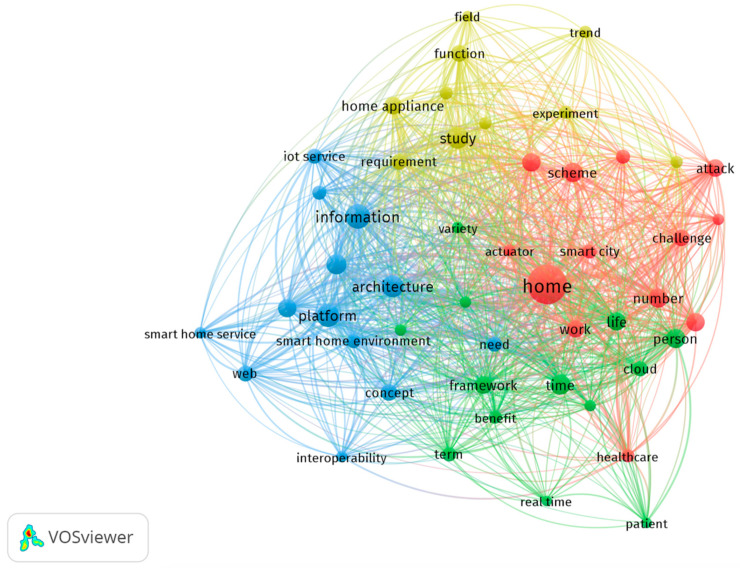
Analysis of co-occurrences in the top 60 keywords.

In this study, we chose all keywords as the unit of analysis, and we set 20 times as the minimum number of occurrences per keyword; therefore, 1997 out of 7693 words met the threshold that was expected to reach the top 100 words. In Figure 8, the co-occurrence analysis of the top 50 keywords is shown. The number of links was 1129 and the total strength of the links was 6439. In cluster one, 13 items were identified. These items had the following names: “actuator”, “algorithm”, “attack”, “challenge”, “communication”, “control”, “healthcare”, “home”, “number”, “researcher”, “scheme”, “smart city”, and “work”. The second cluster encompassed words such as “benefit”, “could”, “framework”, “home automation”, “life”, “patients”, “people”, “real time”, “year”, “smartphone”, “term”, “time”, and “variety” (13 items). A third cluster (13 items) comprised the concepts “architecture”, “concepts”, “implementations”, “information”, “interoperability”, “Internet of Things”, “needs”, “objects”, “platforms”, “smart devices”, “smart home environments”, and “smart home services”. The fourth and final cluster comprised “experiment”, “field”, “function”, “home appliance”, “industry”, “Internet of Things environment”, “development requirements”, “study”, “trend”, and “use” (10 items).

**Figure 9:**
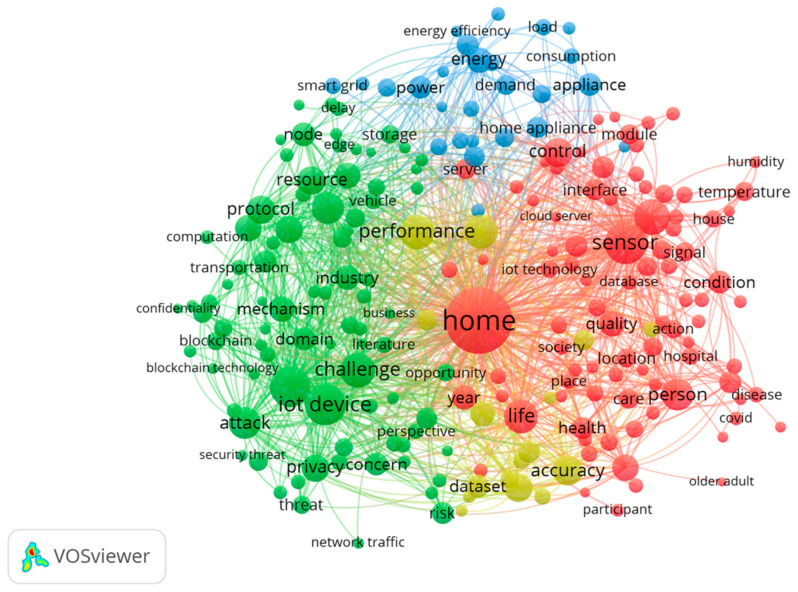
Analysis of the co-occurrences in titles and abstracts.

According to the title and abstract of each publication, Figure 9 illustrates a distribution of themes across all publications in order to provide a better understanding of the themes. Using the title and abstract fields, a full counting method was chosen, where a minimum number of occurrences of a term was fixed at 50 times since the total number of items was 63,077. As can be seen in the map in Figure 9, there were four clusters. The home sensor and its implications are given in the red cluster. It can be concluded that the green cluster represents challenges and risks. The yellow cluster is smaller than all the other clusters, but it was closely associated with all of the other clusters as well. In the blue cluster, there were issues related to energy in the IoT for homes. Research areas identified within the energy field addressed energy-related issues. Increased data transfer rates and the growth of networked devices contribute to higher energy consumption. It is crucial to manage energy consumption, distribution, and production efficiently for the IoT in homes. In accordance with previous studies, scholars mentioned that the increasing demand for energy creates a need to optimize energy consumption in some areas of IoT operations, such as in smart city area [3]. Overall, this study found four research themes with full paper samples, which were “application”, “risk and security”, “data management”, and “energy”.

- Application

The first area of research refers to the potential technological application areas for the IoT in the home. Through features such as the remote control of parameters and home automation, home IoT is able to improve the comfort of living and improve the safety of users. “Temperature”, “light”, and “intensity” were some of the related keywords that showed up. Monitoring a device, controlling it, detecting or simulating its presence, and identifying potential threats were some of the functions. From a smart healthcare perspective, the keyword analysis showed “healthcare”. The application also allows the user to adapt certain functions to suit their personal preferences.

- Risk and Security

Monitoring a device, controlling it, detecting or simulating its presence, and identifying potential threats are some of the related functions. In this cluster, “blockchain”, “privacy”, “authentication”, and “cyber” were major keywords. In the home IoT practices of today, data privacy and security, device security, and network security are key issues to consider. Using a variety of technologies and techniques, such as blockchain technology, cryptography, biometrics, machine learning, data mining, data analytics, and data centers, the researchers suggested that threats can be eliminated or minimized [18].

- Data Management

As a result of networked devices generating large amounts of diverse data, a significant amount of time and effort is required to collect, process, select, and analyze the data generated by these devices. It appears that “cloud server”, “dataset”, and “real-time” were the most frequently used words in this cluster.

- Energy

In line with the rise in the number of networked devices, as well as the increasing speed of data transmission, energy consumption is increasing. It follows that managing and distributing energy efficiently is one of the major challenges that home IoT must face when it comes to energy production, distribution, and consumption. “Demand”, “power”, “energy efficiency”, and “appliance” were in this cluster.

### 4.5. Yearly Trends Themes

As a result of today’s wide availability of technology, such as communication technologies and smart portable devices, the IoT has quickly become one of the most hotly debated topics in every type of industry. Researchers are constantly expanding their scope of research, and their research topics are continuously evolving and deepening. The following analysis allowed us to conclude what the annual trend of studies in the field of IoT in homes was.

#### 4.5.1. In 2018

**Figure 10:**
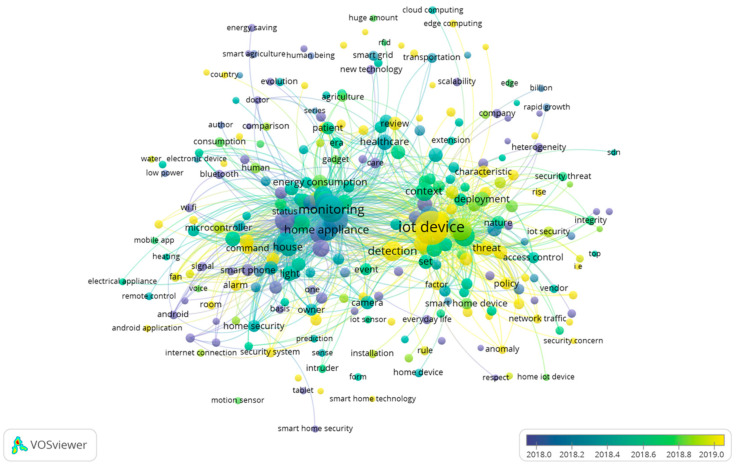
Themes overlay for 2018.

**Figure 11:**
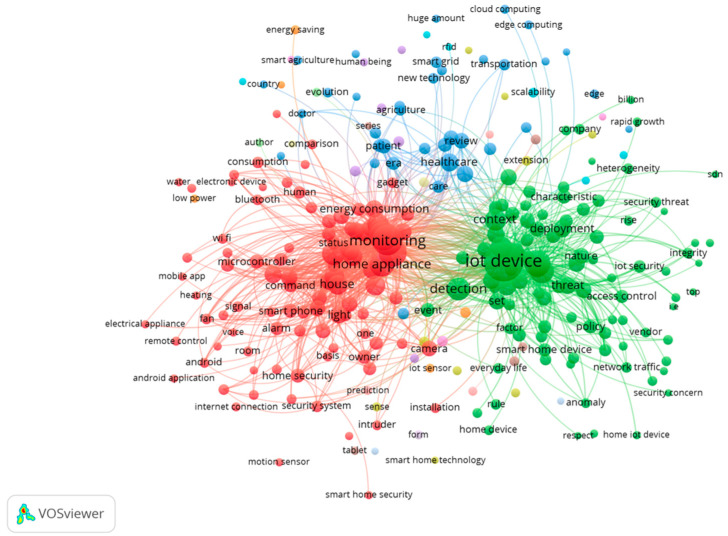
Themes network for 2018.

The research themes for 2018 can be seen in Figure 10 and Figure 11. From the beginning of 2018 to the end of 2018, it seems that the focus of the research shifted from “home appliances and monitoring” to “IoT devices”. As can be seen from Figure 10 below, the research content in 2018 was relatively confined to a single field of interest.

There was a more concentrated research theme for this year, which was “homes IoT technology”, which was led by the keywords “monitoring” and “device”. Various research opportunities should be provided to scholars in both of these fields so that they can conduct a wide range of research. In the blue cluster of keywords, “health” and “care” appeared as keywords, which is worth mentioning.

#### 4.5.2. In 2019

**Figure 12:**
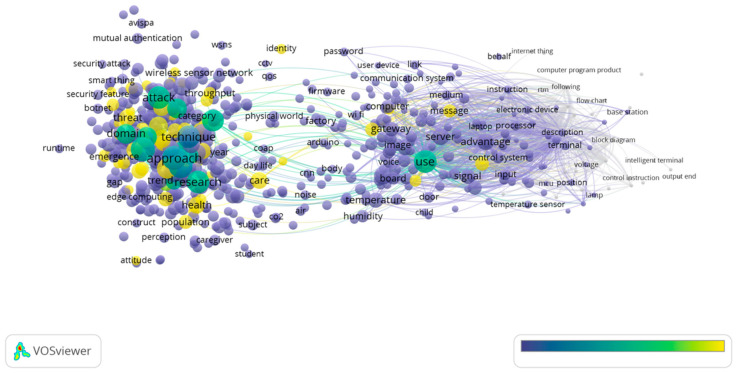
Themes overlay for 2019.

**Figure 13:**
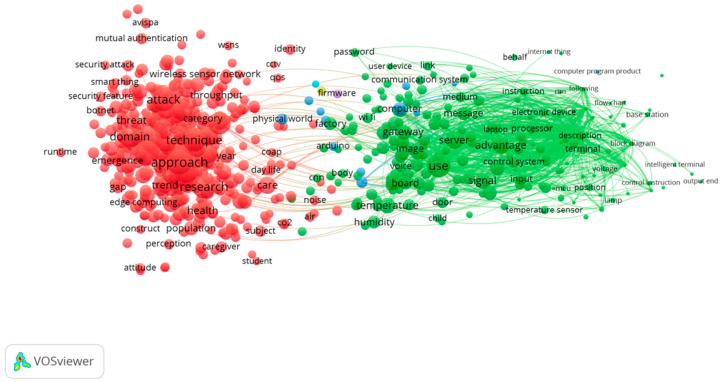
Themes network for 2019.

Research themes for 2019 are shown in Figure 12 and Figure 13. It is important to point out that the topics of research in 2019 seem to be quite clustered, and it is necessary to emphasize again that 2019 was the year that had the most papers published. Regarding this year, it is safe to say that research had reached its zenith. Nevertheless, there were two main types of clustering that scholars studied in terms of their research in 2019. First, there was the red cluster that showed the security and danger that may arise when the IoT is used in a home.

Second, as highlighted in the green cluster of nodes, the IoT in the home showed advantages and provided convenience. Considering the rapid advancement of home IoT in 2019 and scholars’ in-depth research on the positives and negatives of home internet of things, it can be inferred that scholars conducted extensive research on the positives and negatives of it.

#### 4.5.3. In 2020

**Figure 14:**
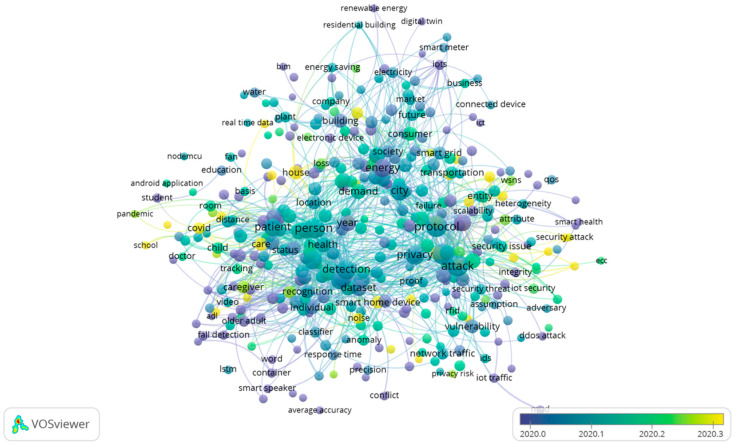
Theme overlay for 2020.

Regarding 2020, 387 items were clustered into 6 groups, namely, “COVID”, “pandemic” “disease”, “distance”, “child”, “school”, and “tracking”; these items are upcoming as a new cluster into the near future that will go with a range of themes. As a result of the mapping analysis, Figure 14 demonstrated that the number of clusters in 2020 has increased compared with the previous two years, demonstrating an increase in the diversity of research. It also indicated that home IoT is being applied in a variety of fields.

Taking a look at the terms, “scheme” had the highest number of occurrences (245), “person” was in second place with 216 occurrences, “patient” was third with 140 occurrences with 1.25 relevance, and “blockchain” was fourth with 113 occurrences and 1.21 relevance. There were also several items that were referred to as home items, such as “house”, “room”, “window”, and “sleep”. These items were co-occurring with the themes around “COVID”. As mentioned before, as people worked or studied “at home” due to the COVID-19 pandemic, a series of descriptive research was published by scholars about the application of the IoT in their homes.

**Figure 15:**
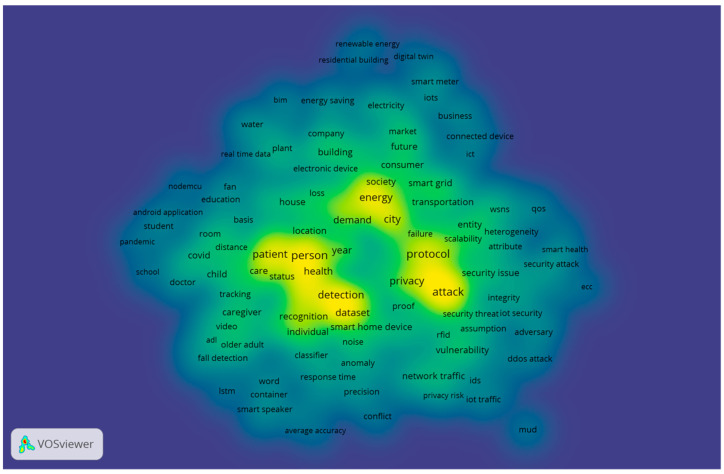
Density visualization 2020.

To better show how the weight of the item is taken into account, Figure 15 gives the cluster density visualization. The ratio of the two colors in densitometry refers to the proportion in which they are mixed based on the total item density of one point, which is derived from equations developed by Van Eck [30]. There is a direct correlation between the color of a point and the color of the background when the total item density of a point is lower. As a way to better illustrate how the weight of the item is taken into account, the cluster density visualization is provided in Figure 15. In the middle-low circle, the specific gravity for “COVID” is shown. A few of the words associated with “COVID” include “room, “school”, “child”, and “distance”.

As a result of the appearance of COVID-19, the core of human existence has been attacked by a pandemic containing a multitude of social and economic consequences. There is no end in sight to its uncontrollable spread throughout the entire world. As per the predictions of the World Health Organization (WHO), the outbreak was expected to peak in June 2020. By utilizing bibliometric analysis, this study investigated and demonstrated the emerging COVID-19 research trends in the field of IoT in homes in a comprehensive manner.

#### 4.5.4. In 2021

**Figure 16:**
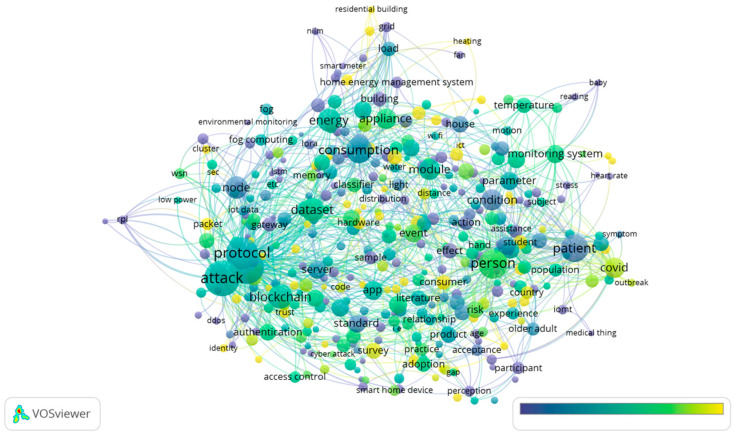
Themes overlay for 2021.

**Figure 17:**
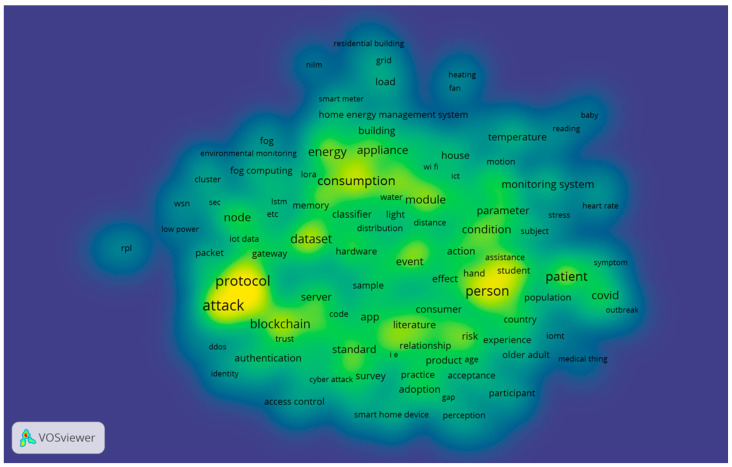
Density visualization for 2020.

Moving to 2021, “attack” had the highest number of occurrences with 320. Normal items with high occurrences were “person” (214), “consumption” (167), “dataset” (136), and “energy” (132). However, what should be emphasized is “COVID” had 109 occurrences with 2.39 relevance, demonstrating its research attraction by scholars. Figure 16 and Figure 17 show the research themes for 2021.

Of 22,305 terms, 673 met the threshold, with the 60% most relevant terms, i.e., 404 terms, shown in Figure 16. In 2021, research related to risk and safety in the IoT in homes was carried out. The item “consumption” was also an important concept addressed, which is also something that we need to pay attention to when it comes to the research area of “energy”. In addition, it is worth mentioning that the theme of “humanity” was highlighted in 2021 by the item “person”.

A visual representation of the cluster density for 2021 can be found in Figure 17. The “protocol” area and the “attack” area were clearly characterized by deep gravity. In the figure below, it is shown to what extent “COVID” and “patients” had different specific gravities. As the grid technology continues to mature, the study of home IoT will also undergo rapid development, which will contribute to the rapid development of this technology in the near future.

#### 4.5.5. In 2022

**Figure 18:**
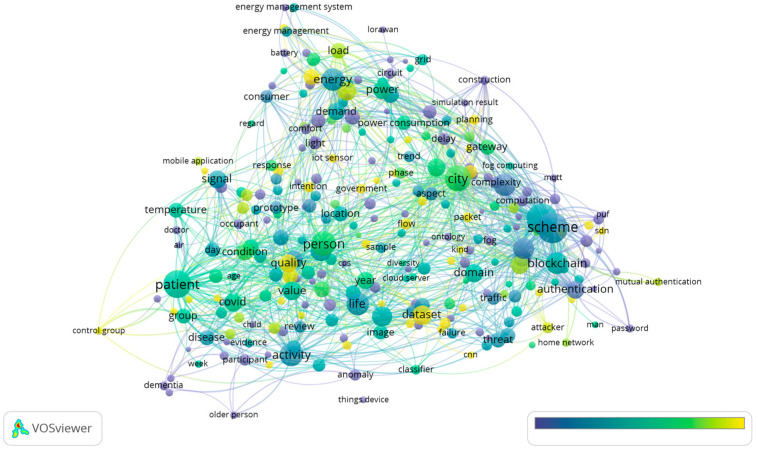
Themes overlay for 2022.

**Figure 19:**
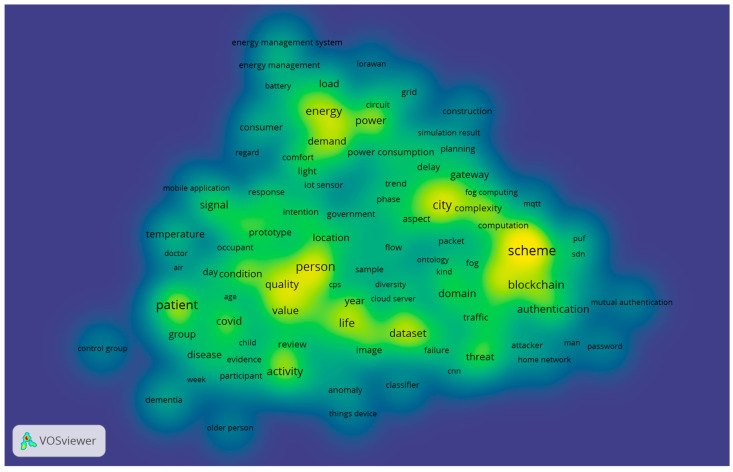
Density visualization for 2022.

As can be seen from Figure 18 and Figure 19, the research theme for home IoT for 2022 is described in more detail. A total of 467 terms met the threshold out of 16,784 terms. In 2022, “home energy management system”, “home network”, and “home environment” were among the terms with high occurrences.

Additionally, the key items of “control group”, “dementia”, and “old people” were clustered together and can be used to justify the fact that the research and application of home IoT are more focused and practical.

COVID-19 was still a concern in the field of home IoT research in 2022. “COVID” (68 occurrences, 1.23 relevance) and “pandemic” (58 occurrences, 1.02 relevance) were closely connected. Furthermore, “home” and “home automation” showed high occurrences and are located in the center.

Moreover, “machine learning model” (15 occurrences, 0.94 relevance), as a critical approach in the science field, also appeared as IoT systems are increasingly relying on machine learning techniques and their potential is being exploited. Additionally, in order to maintain security and privacy in smart systems, there must be mechanisms and regulations in place to do so.

Based on the results presented in Figure 19, it can be seen that the “people” orientation was the main theme that emerged in 2022. These findings are consistent with the results that were published in [31], which revealed that home IoT is congruent with assisted living for the elderly with health monitoring devices. The second main research topic is closely related to the overall topic of “risk and security” in a wide range of aspects.

Taking the Results section as a whole, this study found four themes of research, namely, “application”, “risk and security”, “data management” and “energy” (2018–2022), and a full sample of papers. The analysis of annual publications separately shows that there were two specific research themes. One was attributed to “COVID-19 and health”, while the other was attributed to “people-oriented”.

## 5. Discussion

### 5.1. Summary

There has been a great deal of progress in IoT-related research over the last two decades, with some bridging areas of research emerging. For example, with the IoT, many different aspects of everyday life can be made smarter, from smart health to smart education, from smart buildings to smart industries. Home IoT is considered to cover most of the potential applications of IoT solutions.

The house is an indispensable part of the urban ecosystem; presently, a building is a complex entity with multiple interconnected systems and frameworks, such as lighting, utilities, and security. The complexity of a building increases with its size, and buildings are vulnerable to unsettling disturbances, resulting in potential loss of life and resource safety [32].

First of all, we presented a basic overview of the bibliometrics of the IoT in the home. As a second step, co-authorship and co-occurrence analyses were conducted, and VOSviewer was used to visualize the network of co-authorship and co-occurrences. Finally, based on extracted items from files, VOSviewer was also used to analyze the developments in the IoT industry based on thematic trends. Our research examined bibliometric trends in the IoT in homes from 2018 to 2022 to reveal the current state of the field, assess its mainstream research topics, and identify the emerging challenges for the IoT in homes in the near future. In the past few years, people have been able to maximize their comfort at home by taking advantage of technologies, such as telemedicine, homeowner automation, and ubiquitous computing, to maximize their quality of life. The field of home IoT research has recently undergone a new research trend called “deep learning”.

### 5.2. Contributions and Implications

Taking into consideration the research objectives, which are listed at the beginning of this paper, the contribution of this study to the field can be summarized by stating that there were four significant conclusions made.

The first thing to note is that this study provides an overview of the scientific literature that was recently published about IoT for homes. Remarkable growth was observed in the size of the global IoT market for home appliances. Referring to Figure 3, which shows the research output for the year 2019, one can see that the sheer number of papers published reached its peak; the number of papers has been declining every year since 2019, as shown in linear fitting for publications. In the future, it is expected that the number of researchers will gradually decline. Nevertheless, it is possible that this is just an illusion that is being caused by the intense study of the area and the fixation of proper concepts that may result in a decline in research. There are several experts in this field, as listed in the special issue “Internet of Things for Smart Homes III” of *Sensors*, who made significant contributions to this field [33]. It is clear that there are some segmented research topics that could be considered under the topic of home IoT, such as green communication, monitoring and control, and energy management in homes, but this clarity might subdivide the results. Therefore, further research will involve more specific keyword searches for better forecasting. 

There is no doubt that smart homes and IoT technologies are having a profound impact on various aspects of society. A great deal of research was conducted on home IoT in recent years. The enormous increase in the number of citations can be viewed as evidence of this conclusion, particularly the exponential distribution, which demonstrates the increasing trend of future citations and applications in the future.

Second, it is also worth mentioning that when analyzing contributions, this study took authorship, citations, and geography into account. There is an attractive aspect of this article in that it offers a comprehensive understanding and a clear picture of how IoT technology in the home has evolved over the course of the past five years and how it is anticipated to develop further. It was found that there were similarities between the country-level use of references in the data set analyzed based on the results of the bibliographic coupling analysis. It was evident from the analysis of the co-authorship networks at the regional and country levels that there is a good opportunity for international collaborations to be formed in the area of home IoT research.

Additionally, a third benefit of this study was the fact that it analyzed collaborations in an effort to identify influential organizations and authors. There were several organizations that were involved in various aspects of related research, and in this research set, they were well integrated as a network of co-authors at the organization level, which consisted of several authors and participants.

Furthermore, home IoT research themes from 2018 to 2022 were identified in this study. Moreover, the annual trends in the home IoT market and evaluating their development were also addressed and summarized. On the basis of the overall sample, this study offered a summary of four main research themes, which were “application”, “risks and security”, “data management”, and “energy”. Our annual analysis of the data for each year also revealed these themes to be present in the annual analysis. However, from year to year, the research highlights differed a bit. 

The analysis of annual publications separately showed that there were two specific research themes. One was attributed to “COVID-19 and health”, while the other was attributed to “people-oriented”. As a result of the COVID-19 pandemic, digital technology was increasingly adopted for use in quarantine to study and work at home.

**Figure 20:**
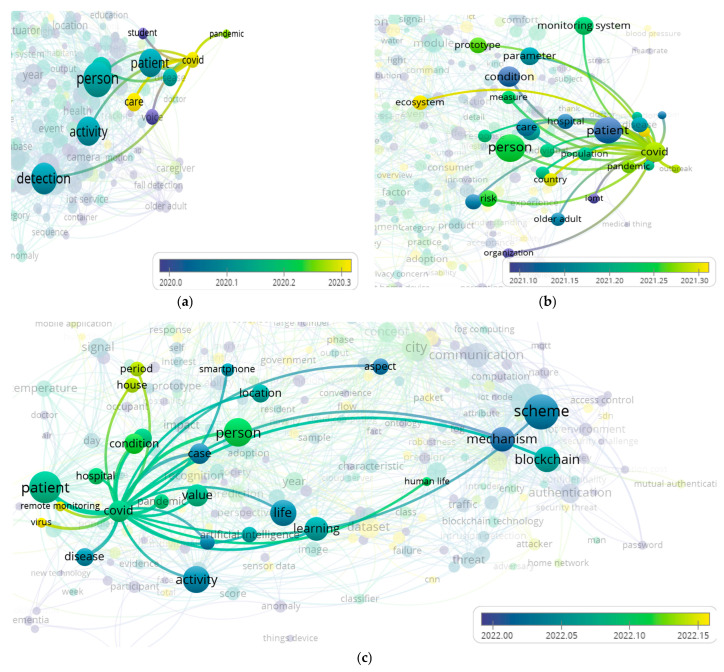
Topics related to COVID-19 in 2020–2022: (**a**) 2020, (**b**) 2021, and (**c**) 2022.

As a result of analyzing the selected relevant articles using the bibliometric networks methods, we were able to analyze themes as they related to the year of the article. It is particularly important to know whether the research topics studied changed due to the impact of COVID-19 on the research. In Figure 20, this study emphasized how COVID-19 overlapped with other clusters and interacted with other items. Taking a look at the figure below, COVID-19 seems to be the topic that generated the largest number of associations in 2022. From 2020 to 2022, it seems that “COVID” became a key item of interest in the research, which appeared at the edge, along with others that are gradually becoming related to other research topics, which indicates that this social phenomenon is having an impact on the home IoT research.

## 6. Conclusions

The purpose of this study was to provide a comprehensive overview of home IoT research in terms of bibliometric analysis and to produce a comprehensive picture of this area in order to enable future scholars to focus on their research effectively and to portray a comprehensive picture of this field. It is also important to acknowledge that there are some limitations to this study that we have to acknowledge.

In conclusion, first of all, the trends in this study were consistent with previous studies, which showed that the number of articles in this field peaked in 2018–2019, coincident with the development of IoT and the expansion of research on the topic [7]. With the aim of applying further research to the main research topic of home IoT, the present study utilized VOSviewer for the formulation of annual research clusters and trends with regard to the primary research topic. Moreover, with this in mind, the main achievement of this research was providing a comprehensive overview of the scientific growth trend and a look at fundamental scientific indicators, publication features, and a visual representation of the home IoT landscape through science maps and network analysis methods. As a result of these research processes, this study suggests a research agenda from a thematic approach based on the findings.

### 6.1. Thematic Agendas

In the process of analyzing and purifying the data, we discovered that related terms such as “home IoT”, ”smart homes”, and “IoT-smart home” [13] were in use by different scholars; however, there is no agreement on the name of the concept. Due to this, it is difficult to clean and filter samples for specific literature reviews, which, in turn, negatively affects the progress of disciplines and applications on a broader scale, as well as the overall growth of the scientific community. The first research agenda we propose is to integrate concepts. In the future, home IoT research should explore the possibilities of a collective and holistic approach to defining IoT applications in the home.

In the field of home IoT, with the exception of one technical issue, our observations led us to conclude that primary research points in the field are the concepts of “humanity” and “people-oriented”. This is consistent with a previous study that showed that it is important to have both relationship-based strategies in a smart home and a technological framework [7,9]. There is a need for more research to be done on home IoT from management, sociology, and anthropology scholars. The second research agenda is the integration of interdisciplinary approaches in the field of home IoT.

### 6.2. Limitations

There are many disciplines that use bibliography analysis to examine the scope of research relating to a specific topic or area of interest. It is important to note that the sample we used for our study was captured from several different databases as part of the research. However, in this study, the databases were not classified and analyzed separately, primarily because there were too many variations in sample sizes. In addition to this, with a sample size of 3880, it may be possible to provide a very comprehensive overview of the research area. However, if we consider the WoS index database, which only provides SSCI results, then it may be possible to extract this information explicitly from it. Furthermore, in order to ensure publication quality, the study restricted the collection of data to only two kinds of publications in order to maintain a high level of quality (conference articles and articles). Future research will extend data collection to other types of publications (e.g., working papers and reviews) to provide more insights and the latest findings in the field in the future. Moreover, Moral-Muñoz et al. (2020) mentioned that the tools for bibliometric research are numbered, and each has its own strengths [16]. Future studies can consider the use of performance analysis tools. Last but not least, with the intention to highlight the annual research changes that occurred as a result of the COVID-19 pandemic, this study focused on research published in 2018–2022; the works of some scholars were focused on several decades of research in certain specific fields, and they were able to establish a historical segmentation of research phases and a future agenda in terms of both theoretical and practical methods based on the analysis of large samples and in-depth analyses [34]. Considering that home IoT is a topic of sustainable development that has been continuously studied for human life and well-being, scholars should give more attention to this topic in order to reflect the complete development process in future research. 

## Figures and Tables

**Table 1:** Review of bibliometric research in the IoT based on the WoS database.

Authors	Article Title	Times Cited *	Included Papers	Utilized Database	Utilized Software
[17] Guo et al. (2021)	A bibliometric analysis and visualization of blockchain	50	3826	WoS Core	CiteSpace, VOSviewer
[15] Sadoughi et al. (2020)	Internet of things in medicine: A systematic mapping study	40	89	WoS	CitNetExplorer
[18] Kamran et al. (2020)	Blockchain and internet of things: A bibliometric study	38	151	WoS Core	Unspecified
[2] Choi et al. (2021)	Smart home and internet of things: A bibliometric study	30	2339	Scopus	Unspecified
[19] Rani et al. (2021)	Security and privacy challenges in the deployment of cyber-physical systems in smart city applications: State-of-art work	5	Unspecified	Scopus	Unspecified
[7] Sun and Li (2021)	A systematic review of the research framework and evolution of smart homes based on the internet of things	5	2874	WoS	CiteSpace
[20] Dai et al. (2021)	A comparative study of Chinese and foreign research on the internet of things in education: Bibliometric analysis and visualization	5	2257	CNKI, WoS	CiteSpace
[21] Uppal et al. (2022)	Fault pattern diagnosis and classification in sensor nodes using fall curve	3	63	Scopus	Unspecified
[22] Leong et al. (2021)	Bibliometric and content analysis of the internet of things research: a social science perspective	3	169	WoS	VOSviewer
[13] Sharm et al. (2021)	RecIoT: A deep insight into IoT-based smart recommender systems	0	90	Scopus	Unspecified

* Extracted on 6 March 2023, WoS core cited.

**Table 2:** Top 10 most active regions, research areas, and organizations of publications.

Subject	Number of Publications	Proportion (%)
**Regions**		
USA	588	15.15
People’s Republic of China	567	14.12
India	465	11.98
South Korea	283	7.29
Saudi Arabia	215	5.54
Italy	205	5.28
England	196	5.05
Pakistan	176	4.55
Canada	140	3.61
Japan	126	3.24
**Research Area (Can Involve Multiple Fields)**		
Computer science	2995	77.19
Engineering	2067	53.27
Telecommunications	1912	49.28
Communication	641	16.52
Mathematics	490	12.68
Instruments and instrumentation	407	10.49
Automation control systems	369	9.41
Mathematical computational biology	343	8.84
Energy fuels	289	7.48
Chemistry	229	5.90
**Organizations**		
National Institute of Technology	59	1.52
Chinese Academy of Sciences	53	1.37
Centre National DE LA recherche Scient fique	47	1.21
Pennsylvania Commonwealth System of Higher Education	40	1.03
University of California	38	0.98
State University of Florida	38	0.98
University of Georgia	36	0.93
Electronics Research Institute Korea	36	0.93
Indian Institute of Technology	35	0.90
University Park	12	0.31

## Data Availability

Data sharing is not applicable to this article as no new data were created or analyzed in this study.
